# Stafne’s bone defect: a systematic review

**DOI:** 10.4317/medoral.25676

**Published:** 2022-12-24

**Authors:** Alini Soares, Luíse Ferreira, Camila Calderipe, Ronell Bologna-Molina, Melissa Damian, Manoela Martins, Felipe Silveira, Ana Carolina Vasconcelos

**Affiliations:** 1 Graduate student, Dental School, Federal University of Pelotas, Brazil; 2 Ph.D., Dental School, University of the Republic, Uruguay; 3 Ph.D., Dental School, Federal University of Pelotas, Brazil; 4 Ph.D., Dental School, Federal University of Rio Grande do Sul, Brazil

## Abstract

**Background:**

This systematic review integrated the available data published in the literature on Stafne’s bone defect (SBD), considering the clinical, imaging and histopathological results.

**Material and Methods:**

An electronic search was undertaken in six databases. Eligibility criteria were: articles in English, Spanish, and Portuguese describing case reports or case series of SBD, reported up to September/2021. Risk of bias was assessed using the Joanna Briggs Institute tool.

**Results:**

A total of 98 articles were retrieved, involving 465 individuals with SBD and were included for quantitative analysis. Mean age was 52.78 years (range: 11-89 years), with male predilection (n=374/80.85%). Radiographs were the most frequent imagiological exams (n=298/64.09%), followed by computed tomography (n=98/21.08%). SBD was more prevalent in the posterior mandible (n=361/93.77%) as a hypodense radiolucent lesion (n=250/77.40%). Mean size was 1.58 cm (range: 0.3-.8.0 cm). Two-hundred-and-two lesions (97.37%) were unilocular and 126 (91.97%) were classified as well-defined. Clinical symptoms were reported in 73 cases, while 68 cases (93.15%) were asymptomatic. Only 34 cases (12.32%) were submitted to histopathological examination. Mean follow-up time was 26.42 ±25.39 months.

**Conclusions:**

SBD is more frequent in male patients in the fifth and sixth decade of life. Classic SBD is radiographically characterized as a single, unilocular and well-defined lesion in the posterior region of the jaw with a radiolucent/hypodense appearance.

** Key words:**Stafne bone defect, stafne, stafne defect.

## Introduction

Stafne’s bone defect (SBD) is a rare lesion, first reported in 1942 by Edward Stafne. The etiology of this condition remains uncertain. It is mostly accepted that SBD is a developmental anatomic impression caused by proliferation or translocation of adjacent structures such as salivary glands or other soft tissues. The incidence ranges from 0.1% to 0.48% in different reports (1) - and the actual incidence may be higher than reported because patients show no abnormal symptoms.

Most of the cases are accidentally observed from radiograph exams during dental treatments. Diagnosis is often made by plain radiography only, but use of more accurate imaging such as computed tomography (CT) scanning, cone-beam CT (CBCT), and magnetic ressonance imaging (MRI) is required for atypical cases (2). Much of the reported cases of SBD have occurred near the angle of the mandible, below the inferior alveolar canal. This defect is most often unilateral and rarely bilateral. Radiographic observation shows round or ovoid well-defined unilocular radiolucency (3). Differential diagnoses include odontogenic or non-odontogenic cystic lesions (4). Since SBD is a benign developmental bony defect, surgical intervention is no longer needed to treat it.

To date, no systematic review summarizing data on SBD has been performed in the literature. Therefore, the objective of this study was to conduct a systematic review of the data available in the literature on SBD in order to answer the question: “What are the general clinical and imagiological features of Stafne's bone defect?” The present research provides information that can improve diagnostic accuracy, allowing general clinicians and surgeons to make informed decisions.

## Material and Methods

- Eligibility criteria

Articles describing case reports, case series or clinical trials of SBD were included. Portuguese, English and Spanish were the selected languages for the included articles. Bibliographic reviews, systematic reviews, editorial reviews, meeting/congress abstracts, experimental studies, *in vitro* or ex vivo studies, and articles in which it was not possible to access the full texts were excluded.

- Infomation sources and search strategies

Electronic searches were carried out in September 2021 in PubMed (National Library of Medicine), Web of Science (Clarivate Analytics), Scopus (Elsevier), Ovid (Wolters Kluwer), Embase (Elsevier), and LILACS (Virtual Health Library) databases. Specific searches were tailored to each database. The retrieved references were exported to the EndNote software (Clarivate Analytics, Philadelphia, USA) and duplicates were removed upon identification.

- Selection process

Titles/abstracts of all references retrieved through the electronic searches were read independently by two review authors. The calibration of the reviewers was verified by assessing agreement among three reviewers (the two review authors and a senior lecturer in Oral Medicine) regarding the evaluation of titles/abstracts of the first 50 references retrieved during the searches. A 0.994 Kappa value demonstrated excellent agreement among reviewers. After calibration, the two reviewers evaluated the references. If the title/abstract met the inclusion criteria, the article was included straight away. The full texts of articles with titles/abstracts with insufficient information for a clear decision were obtained. After evaluation of the full texts, references that met the eligibility criteria were also included. Different opinions with respect to inclusion or exclusion between the reviewers were resolved after discussion with the senior lecturer in Oral Medicine.

- Data extraction and data items

When available, the following data were extracted from each included article and recorded on a standardized form: authors' name and year of publication, country, continent, study design, individual’s sex and age, skin color (white/non-white), imagiological examination modality (radiographis/computed tomography/multiple), imagiological features [number of lesions (single/multiple), location (posterior jaw/anterior jaw), appearance (radiopaque/radiolucent/hypodense/hyperdense), size of lesion (cm), locularity (unilocular/multilocular) and definition (well-defined/ill-defined)]. Presence of clinical symptoms (yes/no), histopathological features (yes/no), and follow up (months).

To establish the location of the lesions, the mandible was classified into: anterior - lesions in the incisor and canine region; posterior - lesions in the premolar/molar/retromolar/regions; anterior and posterior - lesions at both sites. Unavailable data were classified as not informed. Data extraction was carried out by two authors. Discussions with another researcher were also held in order to systematize the data collected.

- Appraisal of the methodological quality of the included studies

The methodological quality of the included articles was assessed using the Joanna Briggs Institute – University of Adelaide tool for case reports or case series (5). The case reports included were assessed according to the following parameters: clear description of the patient’s demographic characteristics, medical history and presentation as a timeline, clear description or presentation of the patient’s current clinical condition, clear description of diagnostic tests and assessment methods, clear description of treatment provided, information on post-intervention clinical condition, identification or report of adverse events, and lessons provided by the case report. The case series included were assessed according to the following parameters: a clear statement of the criteria for inclusion, condition measured in a standard and reliable way, use of valid methods for identification of the condition, consecutive and complete inclusion of participants, clear reporting of the demographics and clinical information of the participants, clear reporting of the outcomes and of the demographic information regarding study site(s)/clinic(s), and use of appropriate statistical analysis. In each article included, the parameters could be rated as “yes” (low risk of bias), “no” (high risk of bias) or “not applicable”.

- Statistical analyses

Statistical analysis was performed using the Statistical Package for the Social Sciences (SPSS) for Windows, version 25.0 (IBM Corporation, Armonk, NY). The mean, standard deviation (SD) and percentages were presented as descriptive statistics.

- Other information

This systematic review was elaborated according to the guidelines of the Preferred Reporting Items for Systematic Reviews and Meta-analyses (PRISMA) Statement (6). A protocol was drafted and registered with the International Prospective Register of Systematic Reviews in Health and Social Care (PROSPERO) of the National Institute for Health Research, UK. The following number was assigned to the systematic review: CRD 42021267994.

## Results

- Study selection

**Figure 1:**
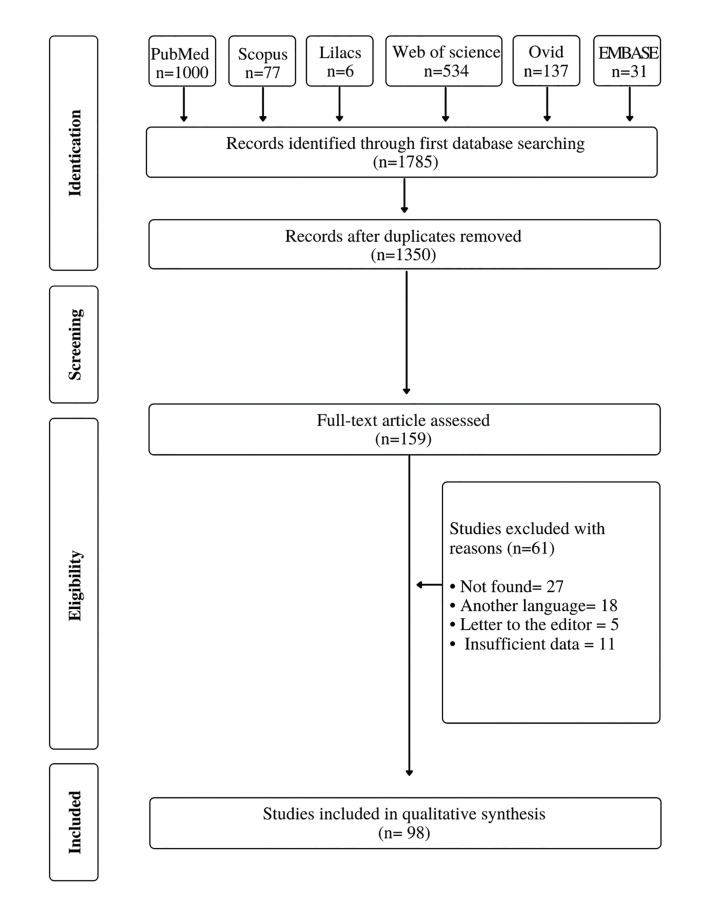
Flowchart of systematic search and study selection strategy.

The search strategy defined for this systematic review through the electronic databases yielded 1785 references. After removal of 435 duplicates, 1,350 references were assessed according to the eligibility criteria. A total of 159 studies were selected for full text evaluation. Of these, 98 fulfilled the eligibility criteria. Therefore, 98 articles reporting 465 cases of clinical and demographic features of SBD were included. Of them, 92 were case reports and 6 were case series. The flowchart depicts the search and the selection process of this systematic review (Fig. 1).

- Demographic and clinical features

Demographic and clinical data are displayed in Table 1. Articles for four continents (26 countries) were included. Most cases were reported in Asia (*n*=189/40.65%), followed by America (*n*=169/36.34%) and Europe (*n*=106/22.89%). Only one case was reported in Oceania (0.21%). Three-hundred-and-seventy-four (80.85%) patients were males and eighty-eight (19.15%) were females. Mean age at diagnosis was 52.78 years (range: 11 to 89 years). Individuals in the sixth decade of life were the most affected (*n*=72/26.09%). Data regarding skin color were available in 11 cases, with 10 affected individuals being white (90.91%) and only one non-white (0.09%). About imagiological examination modality, 298 (64.09%) were radiographs, 98 (21.08%) were CT and 69 (14.84%) were multiple. Regarding imagiological features, 412 (97.40%) lesions were classified as single and 11 (2.60%) were multiple. The posterior jaw was the most affected site, with 361 cases (93.77%). Only 20 cases (6.23%) were in the anterior jaw. Regarding imagiological appearance, 250 (77.40%) exams showed radiolucent lesions and 73 (22.60%) were hypodense. The mean size of the lesions was 1.58 cm (range 0.3-8.0 cm). Two-hundred-and-twenty-two lesions (97.37%) were unilocular and six (2.63%) were multilocular. One-hundred-and- twenty-six (91.97%) lesions were classified as well-defined and 11 (8.03%) as ill-defined. Clinical symptoms were reported in 73 cases, 68 (93.15%) of them being asymptomatic and 5 (6.85%) symptomatic. Only 34 cases (12.32%) were submitted to histopathological examination, with only 22 (6.96%) of them describing the characteristics of the histopathological findings. Follow-up was available for 26 cases, with a mean time of 26.42 ±25.39 months.

- Critical appraisal of included articles

Critical appraisal of the case reports revealed that 78 (98.73%) articles provided a clear description of the patient’s demographic characteristics, with only one having no clear information. Seventy-eight (98.73%) articles provided clear information about patient history and timeline. Details of the current clinical condition of the patients were reported in 76 (96.20%) articles (3/3.80% articles did not report them). Diagnostic tests were clearly described in all included articles. The intervention(s) or treatment procedure(s) were not described in 56 (70.89%) articles and were clearly described in 3/29.11% articles. Post-intervention clinical condition was clearly described in 16 (20.25%) articles (not applied in 56/70.89% articles and not described in 7/8.86% articles). Adverse events (damage) or unforeseen events were identified and described in 12 (15.19%) articles (not applied in 56/70.89% articles and not described in 11/13.92% articles). All articles provided takeaway lessons.

Regarding the critical appraisal of the case series, information on clear criteria for inclusion was provided in all articles. The condition was measured in a standard and reliable way for all participants included. In all cases series, valid methods were used for identification of the condition of the participants included. In all cases, a consecutive and complete inclusion of the participants was also carried out. All case series did provide clear reporting of the demographics of the participants. Four case series (21.05%) did not provide clear clinical information about the participants, while this information was clear in 15/78.95% articles. In four cases (21.05%), outcomes were not clearly reported, where as this information was clear in 15/78.95% articles. The demographic information about the presenting site(s)/clinic(s) was clearly reported in most case series (only 2/10.53% were not reported). Statistical analysis was reported in eight (42.11%) articles (not applied in 11/57.89% articles).

**Table 1:**
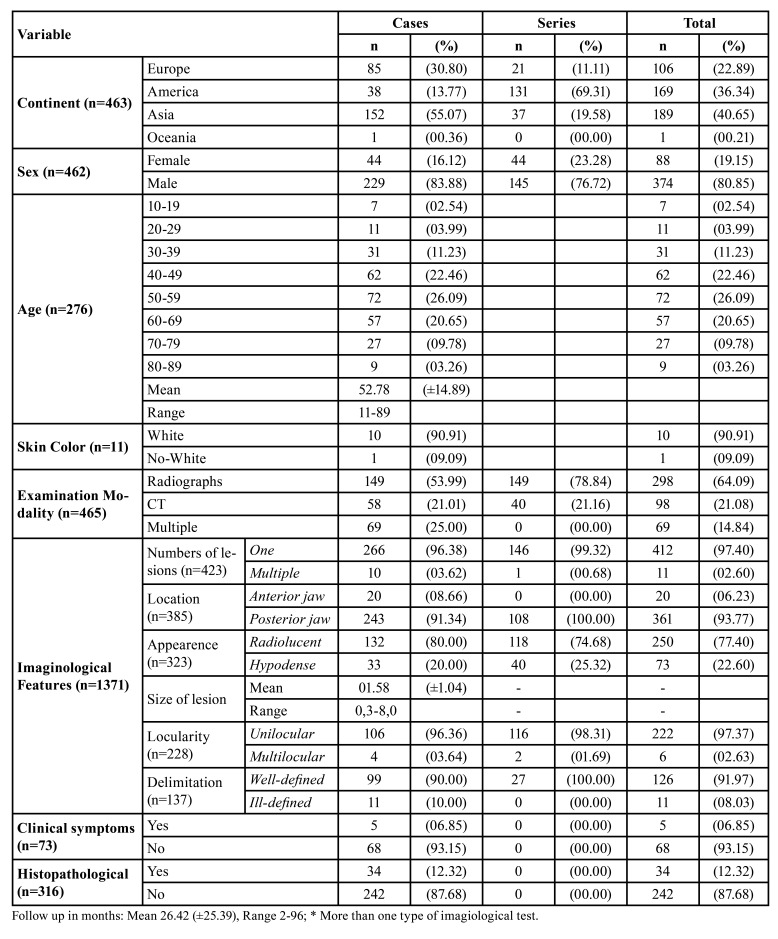
Demographic and clinical characteristics of the sample.

## Discussion

Edward Stafne was the first to describe SBD as “bone cavities situated near the angle of the mandible” (7). The condition has also been described as a static bone cyst, latent bone cyst, idiopathic bone defect, lingual mandibular bone cavity or depression, mandibular salivary gland inclusion, and Stafne bone cyst (8-13). The present systematic review analyzed a total of 465 individuals with SBD from 26 different countries. Most cases were found in Asia and most individuals were white. These results should be evaluated with caution because so far no genetic markers have been found to explain these data. Moreover, China and Japan present a high number of clinical trials publications, especially those related with case reports (14). Moreover, it is important to note that skin color was not reported in most cases, precluding a more precise interpretation.

Although the etiology of SBD is still uncertain, the most popular pathogenesis is a “glandular” hypothesis (2). According to this theory, the lesion originates from compression of the lingual surface of the mandible especially due to the submandibular or sublingual gland, followed by resorption of the lingual cortical plate and finally resulting in a depression or a defect on the lingual aspect of the mandible (15,16). Lello and colleagues (17) proposed the theory that the defect develops as a result of relative ischemia. The authors say that the mandibular lingual cortex is compressed in an area adjacent to the passage of the facial artery and the lesion arises owing to poor blood flow to the cortex due to a compilation of superiorly and medially directed tensile muscle and hemodynamic forces acting on the facial artery, pulling it away from the lingual cortex and thus compromising the nutrition of the cortex.

In this systematic review, SBD showed male predilection at a proportion of 4.25:1. The fifth and sixth decades of life were the most frequently affected, as also observed by Minowa *et al*. (18) and Smith *et al*. (19) in retrospective studies. This finding agrees with data from the literature, which claim a male to female ratio of 4 to 1 (20-22). Curiously, degenerative vascular changes are more commonly encountered in middle-aged males, possibly explaining why SBD is a male phenomenon.

Regarding imagiological aspects, our review shows that radiographs were the most common complementary examination, followed by CT. Typical posterior SBD can be readily diagnosed with a panoramic radiograph due to their unique features. However, CT has additional advantages for the volumetric reproduction of cranium and soft tissues such as the absence of the overlap of anatomic parts that limits the visibility of the structures and the presence of a constant and easily reproducible reference system (23).

Classic SBD was described as a round or ovoid, well-defined, unilocular radiolucency located below the mandibular canal between the first molar and the angle of the mandible (2). In our review, most lesions were single, located in the posterior mandible, with a radiolucent appearance, unilocular and well-defined. Hisatomi *et al*. (24) evaluated 91 panoramic SBD radiographs and observed that the posterior variant was the most frequent (*n*=89/97%). The authors also observed that SBD presented as a unilocular appearance with a round or oval shape. Chen *et al*. (2) reviewed 4000 panoramic radiographs and only found five (0.02%) SBD cases presenting as small, well-defined and radiolucent lesions in the typical first molar to third molar region of the mandible. These data agree with those observed in the present systematic review.

In our review, the size of the lesion ranged from 0.3 to 8 cm, with a mean of 1.58 cm, similar to that observed in other studies (2,11). The differential radiographic diagnosis of SBD includes odontogenic and non-odontogenic well-defined radiolucencies: periapical cyst, traumatic bone cyst, odontogenic keratocyst, dentigerous cyst, fibrous dysplasia, ameloblastoma, and focal osteoporotic bone marrow defect (8,24,25). Liang *et al*. (4) stated that the clue to the correct diagnosis is the characteristic appearance and location of SBD at the mandibular angle below the inferior alveolar nerve canal, whereas odontogenic cysts (periapical cyst, dentigerous cyst) and odontogenic tumors (ameloblastoma) are always present above the inferior alveolar canal.

In the present investigation, clinical symptoms were reported in only five of seventy-three informed cases. In this sense, several studies showed that SBD is accidentally observed on imagiological exams when patients are receiving other dental treatments (4,11,21). Surgical exploration or a biopsy should be performed in atypical cases or other suspected lesions (12). In our review, only 34 cases were submitted to histopathological examination. Most of them revealed the presence of salivary gland tissue, some with many mucous acini and salivary ducts. These findings agree with the origin of SBD development (26-28). Moreover, the low number of histopathological examinations found can be explained by the low number of multilocular and ill-defined lesions (21,29,30).

Since SBD is a benign lesion causing no pathological changes, surgical intervention is no longer needed to treat it. However, follow-ups on a regular basis are recommended to check the possible presence of a tendency to radiographic enlargement or of any abnormal signs of the lesion (4). In our review, mean follow-up was 26.42 months (range: 2-96 months). Follow-up data were provided in only 27 reports. In most cases, no changes were observed in the radiographic appearance and clinical examination. Chaudhry *et al*. (28) have stated that the lesions are not congenital and may show some degree of growth; hence, they are termed as relatively static or latent developmental defects. According to these authors, if the defect is identified as an SBD, a radiograph should be repeated after 12 months to permit the assessment of the dynamics of the process.

The present study has some limitations that should be recognized. First, data were collected across secondary data, and some information was missing or not available. Some clinical data such as skin color, clinical symptoms, and follow-up were not provided in the majority of cases, impairing a more precise interpretation. Second, since diagnosis remains a challenge, the frequency of SBD may have been underestimated. In this sense, it is important to emphasize that the language of publication was restricted to English, Spanish and Portuguese, a fact that may have underestimated the real occurrence of SBDs.

## Conclusions

To our knowledge, this is the first systematic review investigating the main features of SBD. The general dentist, who primarily participates in the diagnostic process, should share diagnostic responsibility with the radiologist, with the two professionals working together to establish a correct diagnostic hypothesis. Also, clinicians should obtain more information about well-defined unilocular radiolucencies - especially those located in the posterior region of the mandible – in order to include SBD in their list of possible diagnostic hypotheses and provide correct management.
